# Nitric Oxide Cooperates With Auxin to Mitigate the Alterations in the Root System Caused by Cadmium and Arsenic

**DOI:** 10.3389/fpls.2020.01182

**Published:** 2020-08-05

**Authors:** Diego Piacentini, Federica Della Rovere, Adriano Sofo, Laura Fattorini, Giuseppina Falasca, Maria Maddalena Altamura

**Affiliations:** ^1^ Department of Environmental Biology, “Sapienza” University of Rome, Rome, Italy; ^2^ Department of European and Mediterranean Cultures: Architecture, Environment, and Cultural Heritage (DICEM), University of Basilicata, Matera, Italy

**Keywords:** arsenic, auxin, cadmium, nitric oxide, *Oryza sativa*, root system

## Abstract

*Oryza sativa* L. is a worldwide food-crop frequently growing in cadmium (Cd)/arsenic (As) polluted soils, with its root-system as the first target of the pollutants. Root-system development involves the establishment of optimal indole-3-acetic acid (IAA) levels, also requiring the conversion of the IAA natural precursor indole-3-butyric acid (IBA) into IAA, causing nitric oxide (NO) formation. Nitric oxide is a stress-signaling molecule. In rice, a negative interaction of Cd or As with endogenous auxin has been demonstrated, as some NO protective effects. However, a synergism between the natural auxins (IAA and/or IBA) and NO was not yet determined and might be important for ameliorating rice metal(oid)-tolerance. With this aim, the stress caused by Cd/As toxicity in the root cells and the possible recovery by either NO or auxins (IAA/IBA) were evaluated after Cd or As (arsenate) exposure, combined or not with the NO-donor compound sodium-nitroprusside (SNP). Root fresh weight, membrane electrolyte leakage, and H_2_O_2_ production were also measured. Moreover, endogenous IAA/IBA contents, transcription-levels of *OsYUCCA1* and *OsASA2* IAA-biosynthetic-genes, and expression of the IAA-influx-carrier *OsAUX1* and the IAA-responsive *DR5::GUS* construct were analyzed, and NO-epifluorescence levels were measured. Results showed that membrane injury by enhanced electrolyte leakage occurred under both pollutants and was reduced by the treatment with SNP only in Cd-presence. By contrast, no membrane injury was caused by either exogenous NO or IAA or IBA. Cd- and As-toxicity also resulted into a decreased root fresh weight, mitigated by the combination of each pollutant with either IAA or IBA. Cd and As decreased the endogenous NO-content, increased H_2_O_2_ formation, and altered auxin biosynthesis, levels and distribution in both adventitious (ARs) and mainly lateral roots (LRs). The SNP-formed NO counteracted the pollutants’ effects on auxin distribution/levels, reduced H_2_O_2_ formation in Cd-presence, and enhanced *AUX1-*expression, mainly in As-presence. Each exogenous auxin, but mainly IBA, combined with Cd or As at 10 µM, mitigated the pollutants’ effects by increasing LR-production and by increasing NO-content in the case of Cd. Altogether, results demonstrate that NO and auxin(s) work together in the rice root system to counteract the specific toxic-effects of each pollutant.

## Introduction

Environmental soil pollution by toxic metal(oid)s is a grave threat to agricultural sustainability and food security, especially in developing and overpopulated countries. Rice (*Oryza sativa* L.) is the staple food of an estimated 3.5 billion people worldwide but also represents one of the main causes of toxic metal poisoning for humans (Abtahi et al., 2017). The heavy metal cadmium (Cd) and the metalloid arsenic (As) are soil contaminants carcinogenic to humans and animals (International Agency for Research on Cancer, 1987; International Agency for Research on Cancer, 1993).

In rice, ionic Cd^+2^ is absorbed by the roots and then rapidly transported to the shoot by xylem loading, reaching the grains after xylem-to-phloem transfer (Uraguchi and Fujiwara, 2012). In Arabidopsis and rice, Cd induces oxidative stress, morphological and developmental changes, and alterations in auxin pathway, ultimately resulting into plant growth inhibition and productivity decline (Fattorini et al., 2017a; Ronzan et al., 2018, and other references therein).

Inorganic arsenate [As(V)] is one of the most toxic and predominant As forms in aerobic environments and, in rice, is taken up by phosphate transporters, reduced to arsenite [As(III)] in the root cells, loaded into the xylem, and finally delivered to the grain through the phloem (Zhao et al., 2010). In addition to causing oxidative stress and lipid peroxidation, As(V) exerts its toxicity especially by replacing inorganic phosphate (Pi) in key biochemical reactions and cellular signaling (Garg and Singla, 2011; Finnegan and Chen, 2012).

Auxins are the plant hormones indispensable for controlling a wide range of developmental processes, under both normal and stress conditions. In root systems, proper levels of indole-3-acetic acid (IAA), the most abundant natural auxin, are required for the formation, development, and maintenance of the roots (Overvoorde et al., 2010). The establishment of optimal IAA levels to support root system development also involves the conversion into IAA of indole-3-butyric acid (IBA), a natural IAA-precursor (Epstein and Ludwig-Müller, 1993; Strader and Bartel, 2011). Although many studies on Arabidopsis mutants defective in IBA-to-IAA conversion suggest that IBA must be converted into IAA to exert auxin activity (Zolman et al., 2008; Strader et al., 2010), evidences have been reported supporting that IBA may act on rice rooting independently of IAA (Chhun et al., 2003; Wang et al., 2003; Chhun et al., 2004). In addition to derive from IBA, IAA is produced in plants by two main biosynthetic pathways, the tryptophan (Trp)-independent and the Trp-dependent pathways (Mano and Nemoto, 2012). In the Trp-dependent pathway, the *α*-subunit of the anthranilate synthase enzyme, which catalyzes the first reaction for Trp-dependent-biosynthesis, is encoded by *ASA1* and *ASA2* genes, while, downstream in the same pathway, the YUCCA (YUC) family of flavin monooxygenases catalyze the conversion of indole-3-piruvic acid (IPA) into IAA (Wang et al., 2018 and references therein). In rice, the IAA-biosynthetic gene *OsASA2* is a defense-activated gene (Tozawa et al., 2001), involved in the plant response to Cd and As (Ronzan et al., 2018) while, among the at least fourteen *YUCCA-like* genes up to now identified, *OsYUCCA1* has been proposed as a key gene for IAA biosynthesis in normal (Yamamoto et al., 2007; Zhang et al., 2018) and during iron-deficiency conditions (Sun et al., 2017).

To efficiently regulate plant development and to convert environmental changes into well-coordinated plant responses, auxin(s) interact(s) in complex networks with other plant hormones and signaling molecules, including nitric oxide (NO) (Freschi, 2013).

Nitric oxide may function both downstream and upstream of auxins, and an increased NO production is known to occur in the roots after exogenous auxin application or in auxin-overproducing mutants in numerous plants, including rice (Chen and Kao, 2012). Moreover, NO production occurs in the conversion of IBA into IAA (Schlicht et al., 2013). By contrast, studies performed in Arabidopsis with the application of NO-specific donors (*e.g.* sodium nitroprusside, SNP), or under stress-conditions, have proven that NO can modulate auxin levels by affecting biosynthesis, degradation, conjugation, distribution, signaling, and enhancing auxin-influx by an increased expression of the *AUXIN-RESISTANT 1* (*AUX1*) gene (Liu et al., 2018). Nitric oxide has been reported to affect rice root growth by regulating auxin transport under nitrate supply (Sun et al., 2018). In addition, several studies have addressed the auxin–NO interaction under metal-stress conditions, however, with contradictory results. The Cd-induced NO-accumulation in *A. thaliana* primary root reduced auxin levels negatively regulating auxin transport and signaling and so negatively affecting root meristem growth (Yuan and Huang, 2016). By contrast, in the same plant under Mg-deficiency, NO positively affected auxin levels by regulating *AUX1* expression (Liu et al., 2018). In *Oryza sativa* seedlings, exogenous NO improved resistance against Hg-induced toxicity by promoting the IAA transport in roots, with this resulting into an increased root growth (Chen Z. et al., 2015); but under Fe-deficiency, NO inhibited root elongation by decreasing auxin levels (Sun et al., 2017).

Taken together, the complex mechanisms underlying the interaction between NO and exogenous/endogenous auxins during the metal/metalloid stress need to be better investigated. In rice, As and Cd damage the root system by altering IAA biosynthesis and distribution (Ronzan et al., 2018), and NO alleviates some effects due to Cd toxicity (Piacentini et al., 2020). However, further efforts should be made to deepen the knowledge about the likely interaction between NO and auxin(s) in rice root system in response to the metal/metalloid in order to ameliorate the stress-tolerance of this important food crop.

To this aim, the stress caused by Cd/As toxicity in the rice fibrous root system formed by embryonic adventitious roots (ARs) and post-embryonic lateral roots (LRs) and lateral root primordia (LRPs) (Rebouillat et al., 2009) and the possible recovery by either NO or auxins (IAA/IBA) were evaluated after Cd or As (arsenate) exposure, combined or not with the NO-donor compound sodium-nitroprusside (SNP). Moreover, endogenous IAA/IBA contents, the transcription-levels of *OsYUCCA1* and *OsASA2* IAA-biosynthetic-genes, and the expression of the IAA-influx-carrier *OsAUX1* and the IAA-responsive *DR5::GUS* construct were analyzed, and NO-epifluorescence levels were measured.

The results showed that the negative effects of Cd and As causing alterations in root NO levels and auxin biosynthesis, levels, distribution, and cellular damages were mitigated by the exogenous application of IBA, IAA, and the NO-donor SNP, demonstrating that NO and auxins work together to counteract the pollutants’ toxicity in the root system of rice.

## Materials and Methods

### Plant Material and Growth Conditions

Seeds of *Oryza sativa* L. ssp. Japonica cv. Nihonmasari (Nihon) (wild type, wt) of *OsDR5::GUS* (Wang et al., 2014) and *OsAUX1::GUS* (Yu et al., 2015) transgenic lines were surface sterilized with ethanol 70% (v/v) for 1.30 min, rinsed three times with ultra-pure water (Milli-Q water), soaked in a solution of 40% (v/v) NaClO for 25 min, and again rinsed three times in sterile Milli-Q water. Then, the seeds were sown in Phytatray-type vessels (Phytatray™ II, Sigma-Aldrich, Saint Louis, USA) containing a half-strength MS (Murashige and Skoog, 1962), 0.1% sucrose and 0.8% agar, at pH 5.6–5.8 (Control medium) and kept for 10 days in long-day conditions (14/10 h light/dark, 210 μmol photons m^−2^s^−1^ and at 27°C).

To the Control medium composition, either 100 µM Na_2_HAsO_4_·7H_2_O (*i.e.*, As) or 100 μM CdSO_4_ (*i.e.*, Cd) was alternatively added and in combination or not with 50 µM of the NO-donor sodium nitroprusside (*i.e.*, SNP) (Sigma-Aldrich, Saint Louis, USA) (Singh et al., 2009). The treatment with SNP at 50 µM was carried out because of its capability to increase intracellular NO levels in this rice cultivar, as verified by epifluorescence analyses and by the use of the NO scavenger compound 2-(4-carboxyphenyl)-4,4,5,5-tetramethylimidazoline-1-oxyl-3-oxide (cPTIO) (Piacentini et al., 2020).

In addition, wt seedlings were also sown on the Control medium with the addition of 1, 10, and 100 μM of IBA or IAA (Sigma-Aldrich, Saint Louis, USA), combined or not with Cd or As (**Supplementary Figure S1**). The concentration of 30 μM of IAA or IBA was also tested, because a lateral root formation similar to 10 μM IAA/IBA was obtained (data not shown); only 10 μM IAA/IBA was used in further experiments. Milli-Q water was used for all culture media.

### Morphological Analysis

After the growing period, 30 wt seedlings grown in the presence of As or Cd alone, or combined with IBA or IAA, or with SNP, were collected and analyzed by measuring root system fresh weight, mean root number, and length of the embryonic adventitious roots (ARs) and mean density of lateral roots (LRs), including lateral root primordia (LRPs) (± SE).

The length of the ARs was measured under a MZ8 stereomicroscope (Leica Microsystems, UK) coupled with AxioCam camera and by processing the images obtained with Zen 2.3 software (Carl Zeiss, Oberkochen, Germany). The LRs and LRPs were counted with a DMRB optical microscope equipped with a DC 500 camera (Leica Microsystems, UK), and the corresponding mean density was expressed as mean number cm^−1^ (± SE). Three independent biological replicates with 30 seedlings each per treatment were carried out with similar results. Data of the second replicate were shown.

### Determination of Electrolyte Leakage and Hydrogen Peroxide

Electrolyte leakage was determined according to the procedure followed by Fu and coworkers (2018) with minor modifications. After 10 days of culture, the root systems of 15 seedlings per treatment grown on the Control medium or treated with 50 µM SNP or 100 µM CdSO_4_ or 100 µM CdSO_4_ +50 µM SNP or 100 µM Na_2_HAsO_4_·7H_2_O or 100 µM Na_2_HAsO_4_·7H_2_O + 50 µM SNP or 10 µM IAA or 10 µM IBA were washed under cold running water and rinsed with deionized water. Then, 0.15 g (fresh weight) of roots per treatment was immersed in 15 ml of deionized water inside autoclavable glass test tubes (V = 28 ml). The tubes were closed with rubber cups and kept at the constant temperature of 32°C for 2 h. After this period, the initial electrical conductivity EC_1_ of each solution was measured. The tubes were then autoclaved at 121°C for 20 min and then left in thermostat until their temperature reached 25°C. Afterwards the final electrical conductivity EC_2_ was measured.

Electrolyte leakage (EL) was calculated using the following formula:

EL (%) = [(EC_1_−EC_0_)/(EC_2_−EC_0_)] × 100, where EC_0_ is the electrical conductivity of deionized water. The measurements were performed with the Delta OHM digital electrical conductivity meter HD 9213-R1 (Delta OHM, PD, Italy). Data were expressed as the average of three technical replicates (N = 5 each) of the same biological replicate. Three independent biological replicates were carried out with similar results; data from the second replicate were shown.

*In situ* detection of hydrogen peroxide (H_2_O_2_) was performed by staining with 3,3-diaminobenzidine (DAB; Sigma-Aldrich) the LRs and LRPs of ten ARs randomly chosen from 30 wt seedlings exposed or not either to Cd, As, SNP, alone or combined, or to IAA or IBA for 10 days. The roots were incubated in a 1% solution of DAB in 10 mM MES buffer (pH 6.5), vacuum-infiltrated for 5min and then incubated at room temperature for 2 h in complete darkness. Then, the reaction was stopped with Milli-Q water. To verify the specificity of the reaction, before the staining with DAB, some roots were treated for 2 h with a solution containing 1mM ascorbate (ASC), a H_2_O_2_ scavenger (Romero-Puertas et al., 2004). Then, the roots were observed under a Leica DMRB optical microscope equipped with a Leica DC 500 camera. Three independent biological replicates with 30 seedlings each per treatment were carried out with similar results. Data of the second replicate were shown.

### GUS Detection Analysis

The root systems of 30 seedlings of either *DR5::GUS* or *AUX1::GUS* transgenic lines grown in the presence of As or Cd, combined or not with SNP, were processed for *β*-glucuronidase (GUS) staining according to Ronzan and co-workers (2018). The roots were cleared with a solution of chloral hydrate/glycerol/water (8:1:2, w/v/v) (Weigel and Glazebrook, 2002) and observed with Nomarski optics applied to a Leica DMRB optical microscope equipped with a Leica DC 500 camera. The image analysis was performed using LEICA IM1000 Image Manager Software. Three independent biological replicates with 30 seedlings each per treatment were carried out with similar results. Data of the second replicate were shown.

### Quantitative RT-PCR Analysis of *OsASA2* and *OsYUCCA1* Genes in Wt Roots

The root system of 10 wt seedlings grown in the presence/absence of Cd or As, combined or not with SNP, was harvested, frozen in liquid nitrogen, and stored at −80 °C prior to RNA extraction. Total RNA was isolated using the Spectrum Plant Total RNA Kit (Sigma-Aldrich, Saint Louis, USA) according to the manufacturer’s instructions and was then quantified using NanoDrop™ One Spectrophotometer (Thermo Fisher Scientific, Inc.).

For cDNA synthesis, 1 μg of total RNA was reversely transcribed using SensiFAST™ Reverse Transcriptase cDNA synthesis Kit (Bioline, London, UK) according to the manufacturer’s instructions.

Relative levels of *OsASA2* and *OsYUCCA1* mRNAs were examined by real-time PCR using LineGene 9620 qPCR detection system (Bioer, China).

Specific primers were designed (**Supplementary Table S1**) using NCBI Primer-BLAST for both the genes of interest. The reference genes used were *OsGAPDH*, *OsUBQ5*, and *OsActin-1* (Almas and Kamrodi, 2018; Ronzan et al., 2018).

The qRT-PCR experiments were carried out in 10 μl reactions containing the final concentration of 250 nM for each primer, 1× SYBR green (SensiMIX™ SYBR^®^ No-ROX Kit, Bioline, London, UK) and 1 μl of a 1:10 cDNA dilution. Amplification parameters were: 95°C for 3 min; 40 amplification cycles (95°C for 15 s, 60°C for 30 s). Data were expressed as the average of three technical replicates of the same biological replicate. Three independent biological replicates were carried out with similar results; data from the second replicate were shown.

### Detection of NO in Wt Roots

Nitric oxide content was determined in 30 roots of wt seedlings, grown on the Control medium or treated with IAA or IBA, alone or combined with Cd or As, or Cd or As taken separately, by using the specific NO fluorescent probe 4-amino-5-methylamino-2′,7′-difluorofluorescein diacetate (DAF-FM DA, Sigma-Aldrich, Saint Louis, MO, USA) (Chen J. et al., 2015). After incubation, the roots were washed three times with fresh 20 mM HEPES/NaOH buffer to remove the excess of the probe and immediately observed under a Leica DMRB optical microscope using filters with excitation at 490 nm and emission at 515 nm, equipped with a Leica DC 500 camera and the relative fluorescence quantified using ImageJ software, V1.52a (https://imagej.nih.gov/ij. RRID : SCR_003070). Three independent biological replicates with 30 roots each per treatment were carried out with similar results. Data of the second replicate were shown.

### Auxin Quantification

The extraction of IAA and IBA was carried out on 300 mg-aliquots of roots of wt seedlings not exposed or exposed to Cd or As, combined or not with SNP, and ground into powder with liquid nitrogen. To each sample, 3.0 ml of extraction solvent (2-propanol/H_2_O/HCl 37%; 2:1:0.002, v/v/v) was added. The tubes were shaken at a speed of 150 rpm for 20 min at 4°C. To each tube, 3.0 ml of dichloromethane was added. Then, the samples were shaken for 30 min at 4°C and centrifuged at 15,000 g for 5 min. After centrifugation, 1.0 ml of the solvent from the lower phase was transferred into a screw-cap vial, and the solvent mixture was concentrated using an evaporator with nitrogen flow. Finally, the samples were re-dissolved in 60 μl methanol and stored at −20°C before quantitative analysis. The quantitative determinations of IAA and IBA were carried out by high-performance liquid chromatography coupled with mass spectrometry, according to Veloccia and co-workers (2016). Pure standards of IAA and IBA were used for quantification (Duchefa Biochemie B.V., Haarlem, The Netherlands). The internal standards used were [^2^H_5_] IAA and [^2^H_9_] IBA (OlChemIm Ltd, Olomouc, Czech Republic; crystalline form, purity > 97% for HPLC). Data were expressed as the average of three technical replicates of the same biological replicate. Three independent biological replicates were carried out with similar results; data from the second replicate were shown.

### Statistical Analysis

Statistical analysis was performed using one-way ANOVA test followed by Tukey’s post-test (at least at P < 0.05) through GraphPad Prism 8.0 software. All the experiments were performed in three biological replicates as detailed in each paragraph.

## Results

### Cadmium and Arsenic Cause Membrane Injury and Hydrogen Peroxide Formation in the Roots, but Exogenous NO Counteracts These Stress-Effects Only in the Presence of Cd

**Figure 1:**
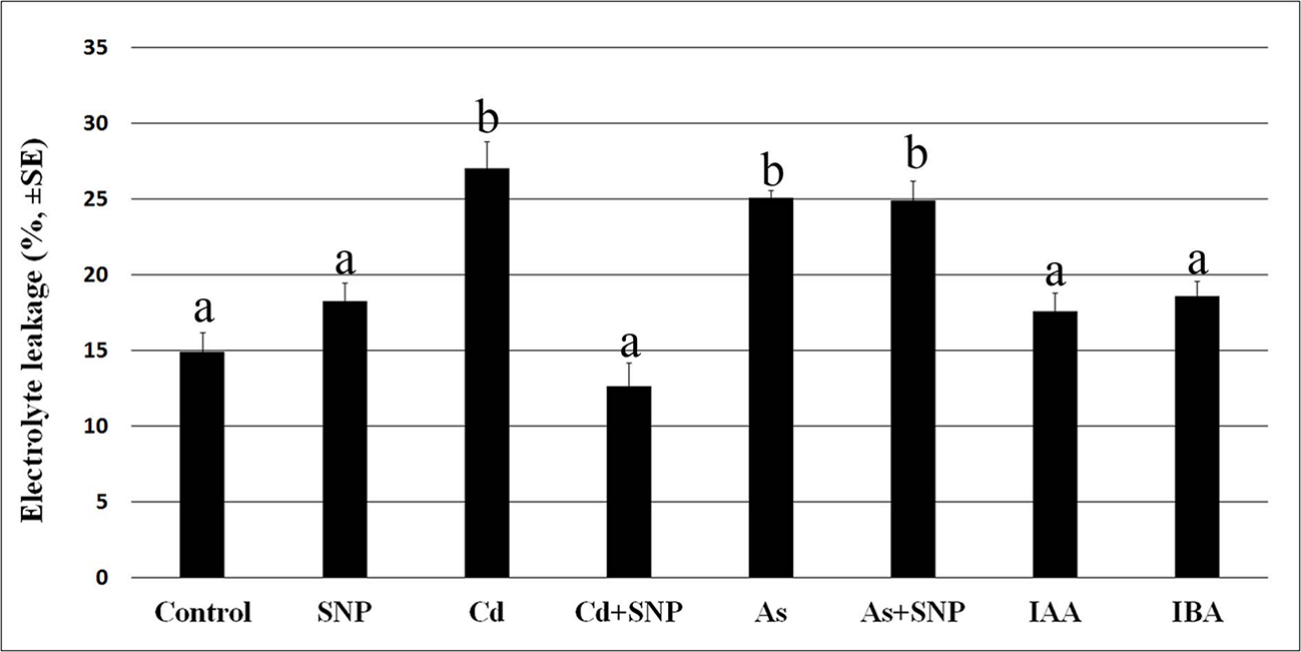
Electrolyte leakage (%, ± SE) in roots of wt rice seedlings not exposed (Control) or exposed to 50 µM sodium nitroprusside (SNP) or 100 μM CdSO_4_ (Cd) alone or combined with SNP, or 100 µM Na_2_HAsO_4_·7H_2_O (As) alone or combined with SNP or 10 μM IAA (IAA) or 10 μM IBA (IBA). Different letters show significant differences among treatments for at least P < 0.05. Columns followed by the same letter are not significantly different. Average of three technical replicates (N = 5 each).

Cadmium and arsenic effects on membrane integrity are known as an expression of their toxicity at cellular level, with membrane injury usually resulting from altered antioxidant defence system. The root system is the first plant part to become in contact with soil pollutants. Thus, we firstly verified the possible membrane injury caused by the selected concentrations of Cd and As, combined or not with the NO-donor SNP, on the rice root system dissected by 10-days-old seedlings of cv. Nihonmasari (wild type). The root cells showed a membrane electrolyte leakage comparable in the Control in comparison with the SNP treatment, with a value possibly consequence of the dissection-stress. By contrast, Cd or As highly and similarly increased the solute leakage (**Figure 1**). When Cd was combined with SNP, the leakage was strongly reduced, but this did not occur in the presence of As plus SNP, showing a pollutant-dependent difference in the exogenous NO capabilities to restore the membrane integrity (**Figure 1**). Neither of the two exogenous auxins (IBA or IAA) at the same concentration caused damage to membranes in addition to that caused by the root system dissection from the seedling because the values of the electrolyte leakage were not significantly different from the Control treatment (**Figure 1**).

**Figure 2:**
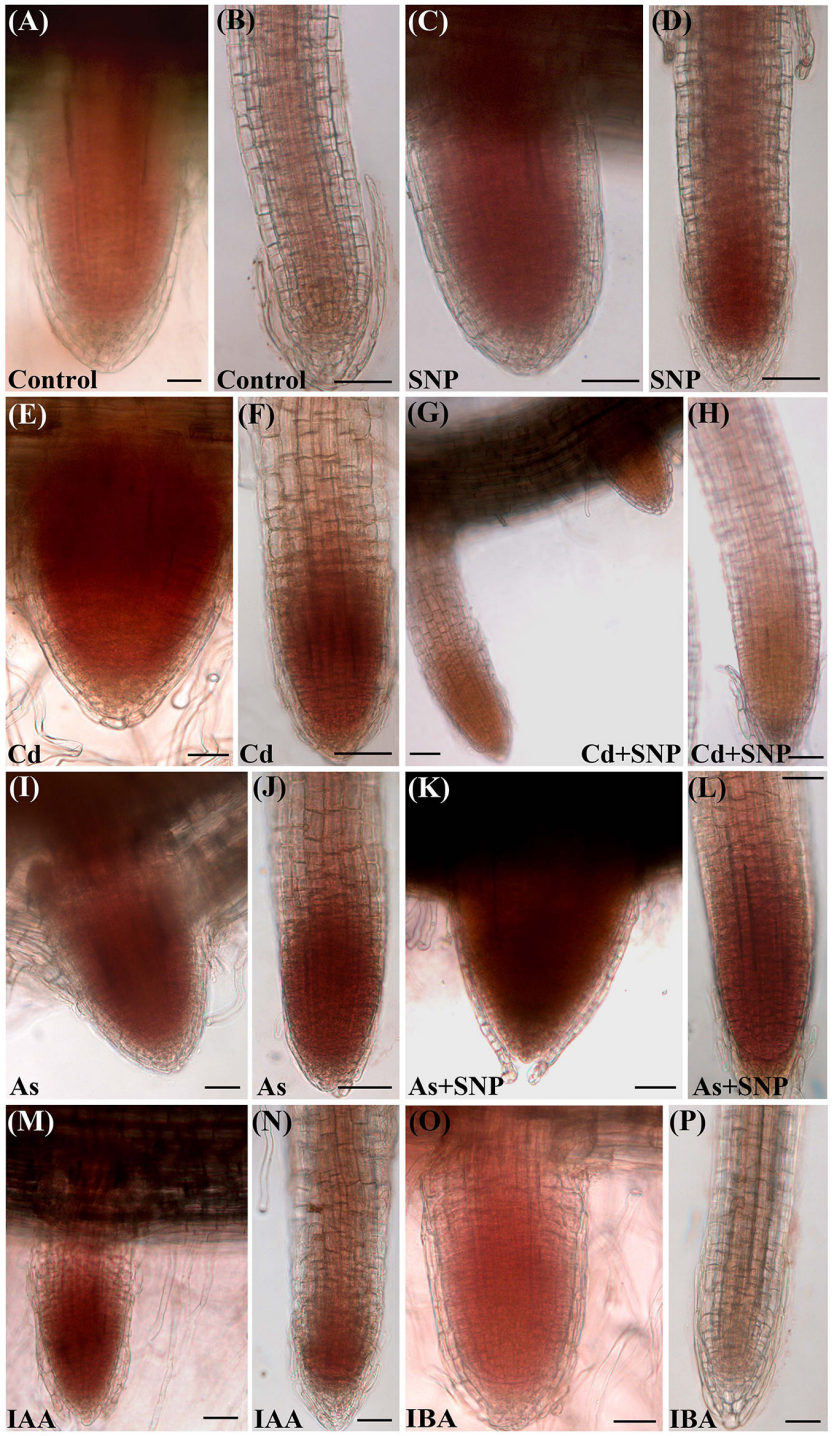
Histochemical detection of H_2_O_2_ by 3,3-diaminobenzidine (DAB) staining in lateral root primordia **(A, C, E, G, I, K, M, O)** and lateral roots **(B, D, F, H, J, L, N, P)** from seedlings not treated (Control) or treated with either 50 µM Sodium nitroprusside (SNP) or 100 μM CdSO_4_ (Cd) or 100 µM Na_2_HAsO_4_·7H_2_O (As), alone or combined, or treated with 10 μM IAA (IAA) or 10 μM IBA (IBA) for 10 days. Bars = 30 µm.

The oxidative damage caused by each pollutant was assessed in the root system as H_2_O_2_ formation monitored by the staining with 3,3-diaminobenzidine (DAB) (**Figure 2**). The specificity of the reaction was verified using ascorbate (ASC), a known H_2_O_2_ scavenger, before the staining with DAB. Results showed that ASC highly counteracted H_2_O_2_ formation independently of the treatment (data not shown).

The production of H_2_O_2_ was observed, as brick red color, mainly in the apices of the LRs and LRPs but with differences between the two root types. In fact, H_2_O_2_ was shown by the entire LRPs independently on the treatment (**Figures 2A, C, E, G, I, K, M, O**), whereas it was restricted to the apex of the elongating/elongated LRs in the treatments with Cd or As and at similar and high levels (**Figures 2F, J**). The production of H_2_O_2_ was low all over in the LRs of the Control treatment (**Figure 2B**), but exogenous NO strongly reinforced it in the LR apex (**Figure 2D**), possibly as consequence of a cell suffering due to a too high NO level in the LR. However, when SNP was combined with Cd, the levels of H_2_O_2_ strongly decreased (**Figures 2G, H**), suggesting a stress-reduction due to the NO derived by SNP in the presence of this pollutant. No similar effect occurred when the NO-donor was combined with As because the production of H_2_O_2_ remained high as with As alone (**Figures 2L, J**, in comparison). Minor differences in H_2_O_2_ formation occurred in the LRs of the treatments with the exogenous auxins in comparison with the Control (**Figures 2N, P**).

### Exogenous IAA and IBA Counteract the Reduction in Lateral Root Formation Caused by the Pollutants With IBA More Efficient Than IAA in a Dose-Dependent Manner

**Figure 3:**
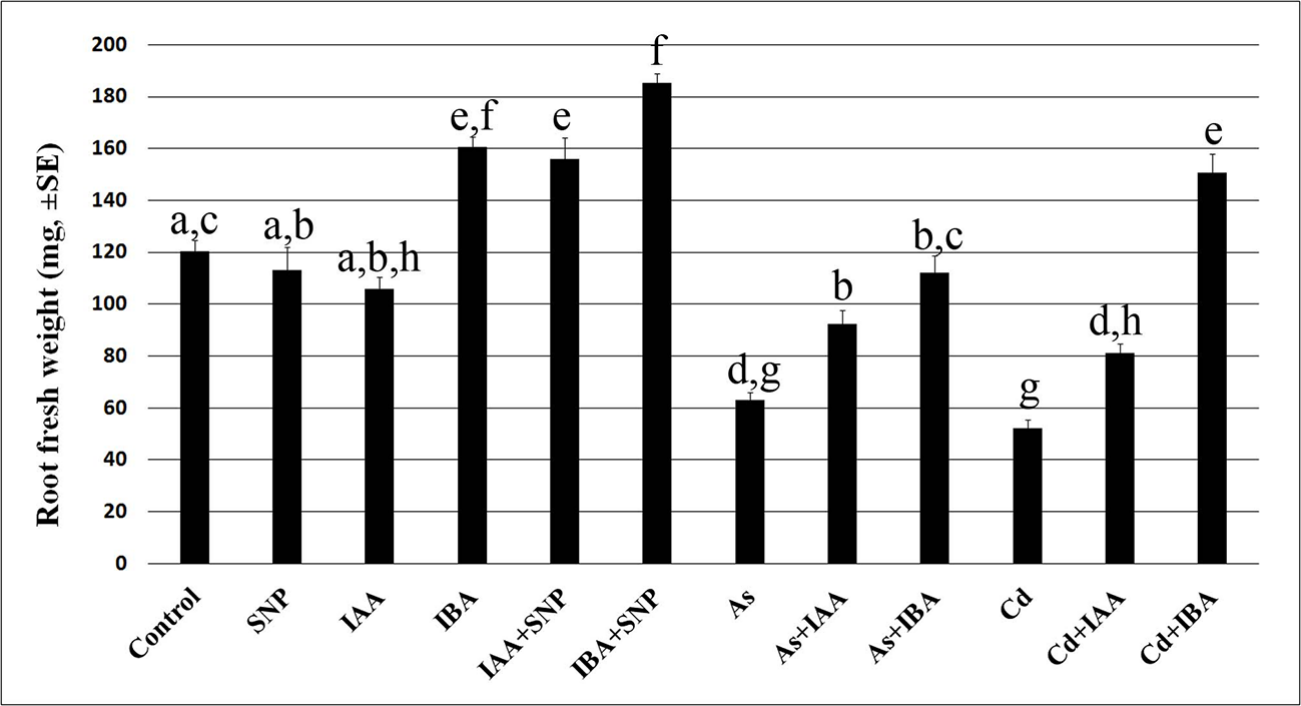
Root mean fresh weight (± SE) in wt seedlings not exposed (Control) or exposed to 50 µM sodium nitroprusside (SNP) or 10 μM IAA (IAA) or 10 μM IBA (IBA), alone or combined with SNP, or 100 µM Na_2_ HAsO_4_·7H_2_O (As) or 100 μM CdSO_4_ (Cd), alone or combined with IAA or IBA. Different letters show significant differences among treatments for at least P < 0.05. Columns followed by the same letter are not significantly different. N = 30.

**Figure 4:**
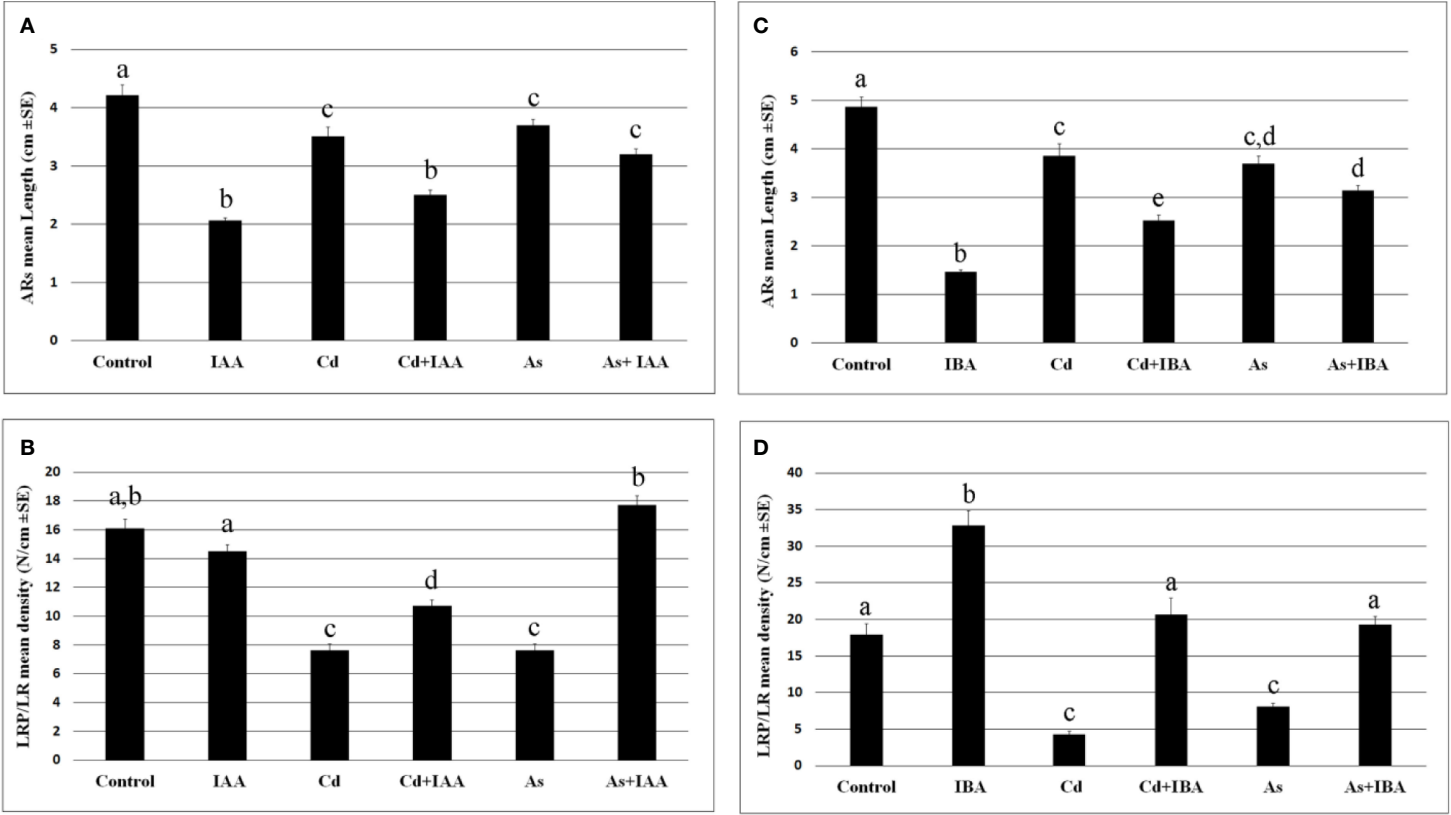
Mean values (± SE) of rice adventitious root (AR) length **(A, C)**, density of lateral root primordia (LRPs) and elongated lateral roots (LRs) **(B, D)** in wt seedlings not exposed (Control) or exposed to 100 μM CdSO_4_ (Cd) or 100 µM Na_2_HAsO_4_·7H_2_O (As) alone or combined with 10 μM IAA (IAA) or 10 μM IBA (IBA) for 10 days. Different letters among different treatments show significant differences for at least P < 0.05. Columns followed by the same letter are not significantly different. N = 30.

The exogenous IAA did not cause significant changes in the root system fresh weight in comparison with the Control, whereas IBA increased it significantly (**Figure 3**), showing different effects of the two auxins on the root system growth, when applied at the same concentration of 10 µM. However, both auxins reduced the AR-length and more than each pollutant (**Figures 4A, C**), but IBA also enhanced LR formation even if many roots remained at the primordium stage (**Figure 4D**). By contrast, each pollutant highly reduced the total fresh weight and in a similar way (**Figure 3**) and the LRP/LR density, in favor of the LRPs, in comparison with the Control treatment (**Figures 4B, D**). When IBA was combined with each pollutant, there was a total recovery of the root fresh weight in comparison with the pollutant alone up to values not significantly different from the IBA alone, at least in the case of Cd (**Figure 3**). The recovery caused by IBA combined with each pollutant was higher than that caused by IAA+ Cd/As (**Figure 3**). However, the LR/LRP density decreased significantly in the presence of IBA combined with each pollutant in comparison with IBA alone, reaching values comparable with the Control (**Figure 4D**) and with more LRPs than LRs (data not shown). Differently from IBA, the exogenous IAA combined with Cd caused a significant reduction in LRP/LR density in comparison with the Control and IAA alone (**Figure 4B**), whereas when IAA was combined with As, LRPs/LRs density increased up to the Control values. However, in the last treatment, differently from the Control, almost only LRPs were formed. The increase in LRPs/LRs density under IAA + As coupled with an increase in root fresh weight up to values not significantly different from IAA alone (**Figures 3** and **4B**).

When each auxin was applied at a concentration ten-fold lower or higher (1 and 100 µM), LRP/LR formation decreased in comparison with 10 µM, and the combination with each pollutant resulted into a further, and significant, rooting-decrease at both 1 and 100 µM auxin concentrations. The strongest decrease occurred at the highest concentration for each auxin and pollutant, highlighting that the capability of the exogenous auxins to mitigate the pollutant-induced reduction in rooting is dose-dependent (**Supplementary Figure S1**).

### Exogenous IAA and IBA Counteract the Reduction of NO Levels Caused by Cd, but Are Unable to Counteract That Caused by As

**Figure 5:**
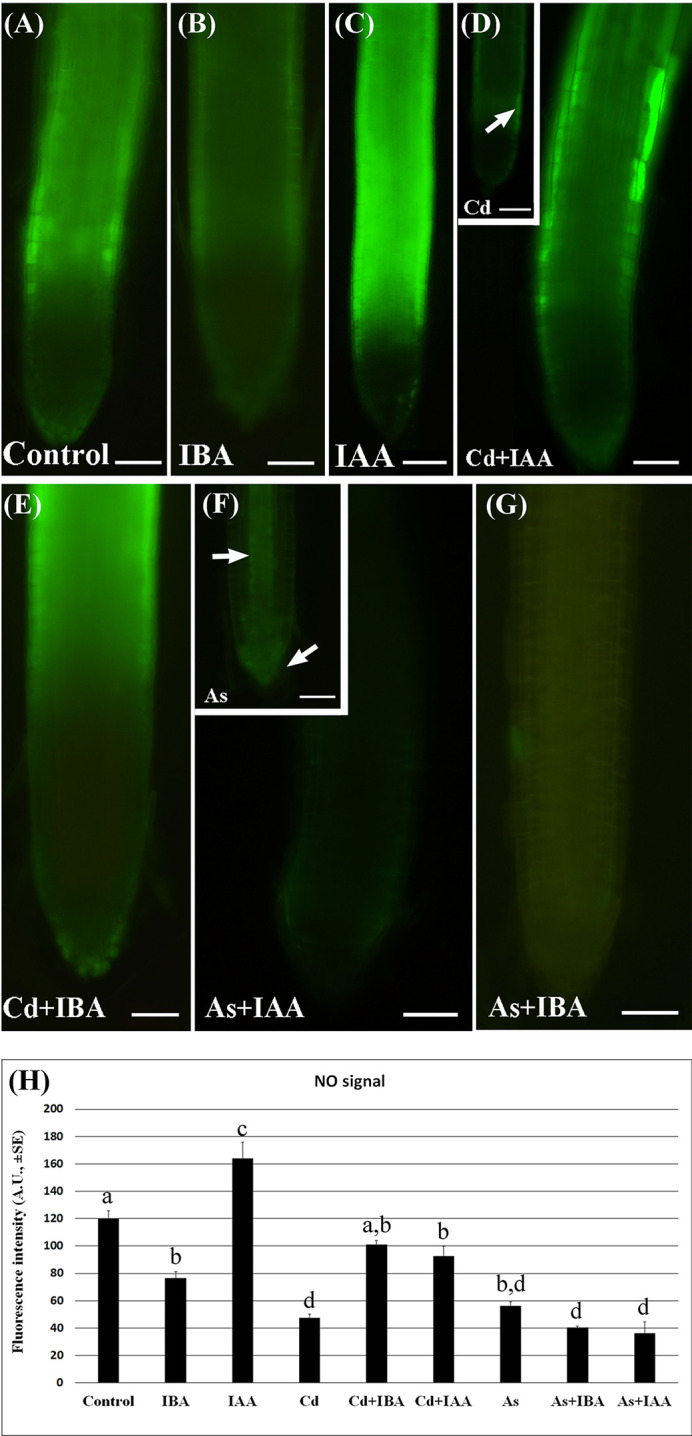
Nitric oxide (NO) epifluorescence signal **(A–G)** in rice lateral roots (LRs) taken from 10-days-old seedlings loaded with 4-amino-5-aminomethil-2′.7′-difluorofluorescein diacetate (DAF-FM DA) and its quantification **(H)**. **(A–G)**, NO signal in bright green color in lateral roots (LRs) not treated (Control) or treated with 10 μM IAA (IAA) or 10 μM IBA (IBA), alone or combined with 100 μM CdSO_4_ (Cd) or 100 µM Na_2_HAsO_4_·7H_2_O (As). Insets in **(D, F)** show LRs treated with Cd or As alone. Bars = 40 µm. **(H)**, mean values of NO fluorescence intensity (± SE) in LRPs/LRs expressed in arbitrary units (A.Us). Different letters among different treatments show significant differences for at least P < 0.05. Columns followed by the same letter are not significantly different. N = 30.

The epifluorescence analysis of the adventitious roots (ARs) loaded with DAF-FM DA revealed the presence of NO in the apex including the cap cells and weakly in the elongation zone, without significant differences between Cd and As, but with a more apical localization in the Control (**Supplementary Figure S2**). By contrast, there was a low NO signal in the apex of the LRs under all treatments (**Figures 5A–G**), whereas the elongation zone showed a high signal in the Control and IAA-treatment and a lower signal under IBA-treatment (**Figures 5A–C**). The NO signal was weak also in the LRs treated with Cd or As alone (**Figures 5D, F**, insets) but with a different localization, *i.e.* differentiating rhizodermis in Cd presence (arrow in **Figure 5D** inset), and apical meristem and forming vasculature in As presence (arrows in **Figure 5F**, inset). The NO signal was reinforced by Cd applied together with either IAA (**Figure 5D**) or IBA (**Figure 5E**) and mainly in the most peripheral tissues. By contrast, the NO signal was highly reduced all over in the LR by the combined application of As with either auxins (**Figures 5F, G**).

The quantification of the epifluorescence intensity confirmed that, compared to the Control treatment, both Cd and As strongly (P < 0.001) reduced the NO signal, while the exogenous auxin treatments differently modulated it. In fact, IAA significantly increased the signal, whereas the IBA significantly decreased it in comparison with Control levels (**Figure 5H**).

Interestingly, both the combined treatments of IBA or IAA with Cd similarly enhanced the level of the NO signal up to values not far from the Control, whereas both the treatments of the two auxins with As similarly and significantly inhibited the signal in comparison with the Control (**Figure 5H**).

### Exogenous NO Mitigates the Effects of Cd and As on Endogenous Auxin Levels and Changes the Expression of the Auxin Biosynthetic Genes

**Figure 6:**
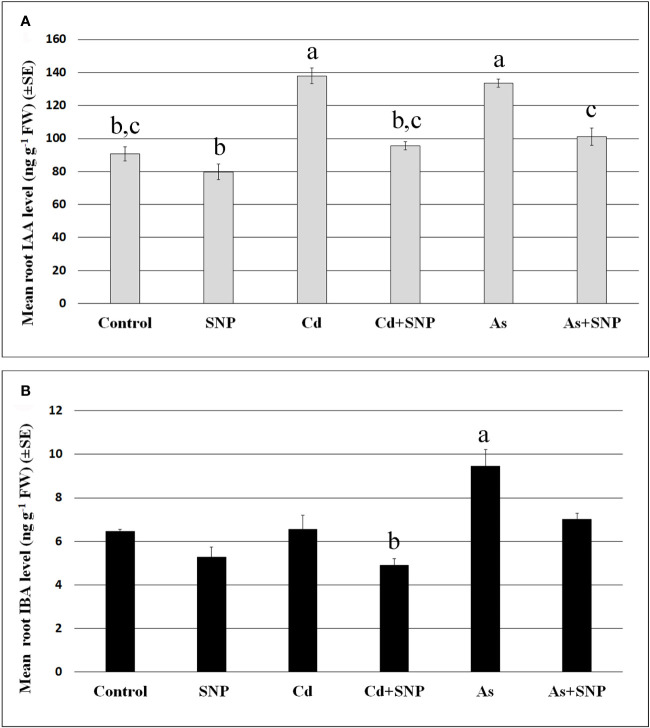
Mean values (± SE) of IAA **(A)** and IBA **(B)** levels (ng g^−1^ FW) in roots of wt rice seedlings not exposed (Control) or exposed to 100 μM CdSO_4_ (Cd) or 100 µM Na_2_HAsO_4_·7H_2_O (As) alone or combined with 50 µM sodium nitroprusside (SNP). Different letters show statistical differences for at least P < 0.05 among treatments. Columns followed by no letter or the same letter are not significantly different. Means are from three technical replicates.

The mean levels of IAA and IBA were analyzed in the roots (**Figure 6**) of the wt seedlings after exposure to the pollutants, combined or not with SNP.

The results show that the mean levels of IAA were significantly increased after Cd or As treatments (**Figure 6A**), while IBA levels, much lower than IAA levels, increased significantly only after As exposure (**Figure 6B**). In addition, the treatment with the NO-donor did not change IAA or IBA levels compared to the Control-treatment (**Figures 6A, B**). By contrast, when SNP was applied together with each pollutant, the over-production of NO resulted in a reduction in the levels of both auxins in comparison with the pollutant alone (**Figures 6A, B**).

Based on these results, further analyses were focussed on the evaluation of the expression of *OsYUCCA1* and *OsASA2* auxin biosynthetic genes under exposure to Cd or As, with/without SNP.

**Figure 7:**
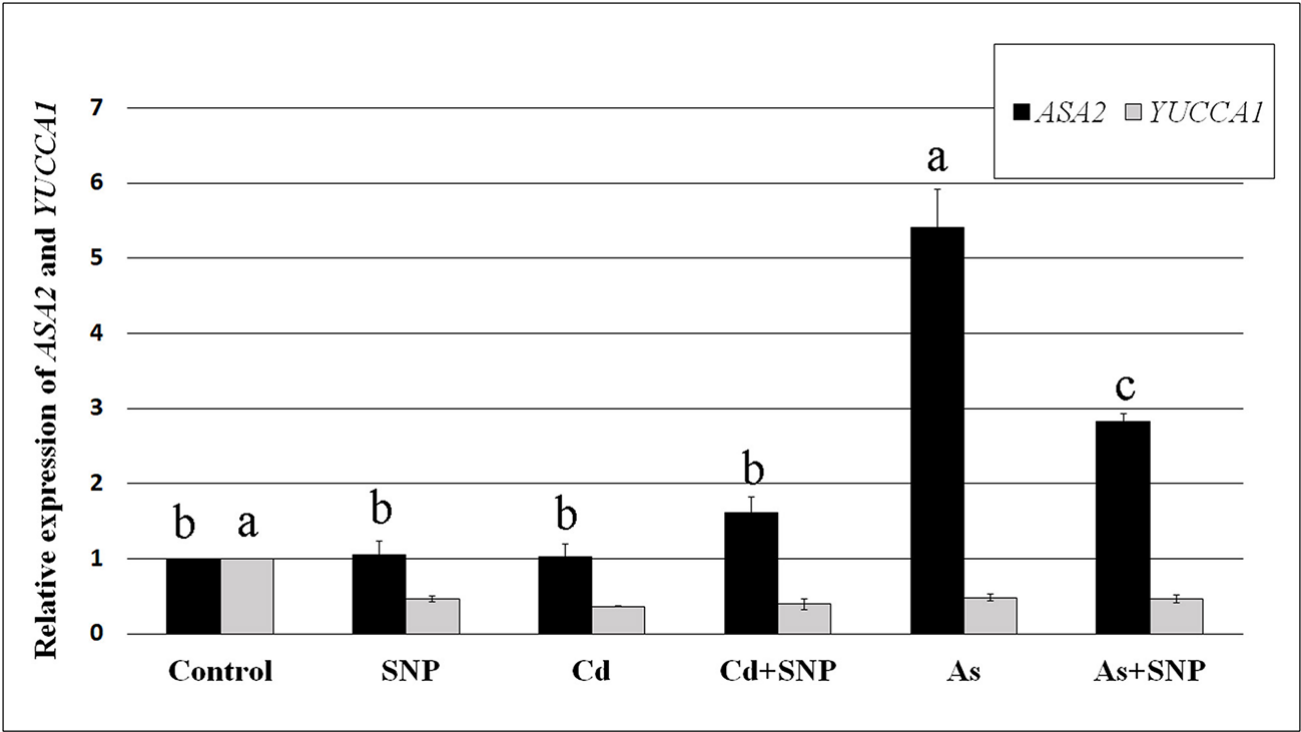
Relative expression of *OsASA2* and *OsYUCCA1* genes (RT-qPCR analysis) (± SE) in wt rice roots not exposed (Control) or exposed to 100 μM CdSO_4_ (Cd) or 100 µM Na_2_HAsO_4_·7H_2_O (As) alone or combined with 50 µM sodium nitroprusside (SNP). The expression levels of the two genes in the Control were set to 1. Different letters show statistical differences for at least P < 0.01 for the same gene among treatments. Columns followed by no letter or the same letter are not significantly different. Means are from three technical replicates.

Both Cd and As significantly (P < 0.001) reduced the expression of *OsYUCCA1*, whereas only As changed *OsASA2* expression, causing an about five-fold (P < 0.001) increase in expression in comparison with the Control-treatment (**Figure 7**). Compared to the Control, the treatments with SNP, alone or combined with the pollutants, reduced (P < 0.001) *OsYUCCA1* expression. By contrast, *OsASA2* expression was not affected by the exposure with SNP alone or supplemented with Cd. However, the As-induced increase in *OsASA2* transcription was reduced about half when the NO-donor was combined with the metalloid, remaining however, significantly (P < 0.01) higher than the Control and the Cd ± SNP values (**Figure 7**).

### Exogenous NO Reduces the Alterations in Auxin Distribution Caused by Cd and As in the Roots

**Figure 8:**
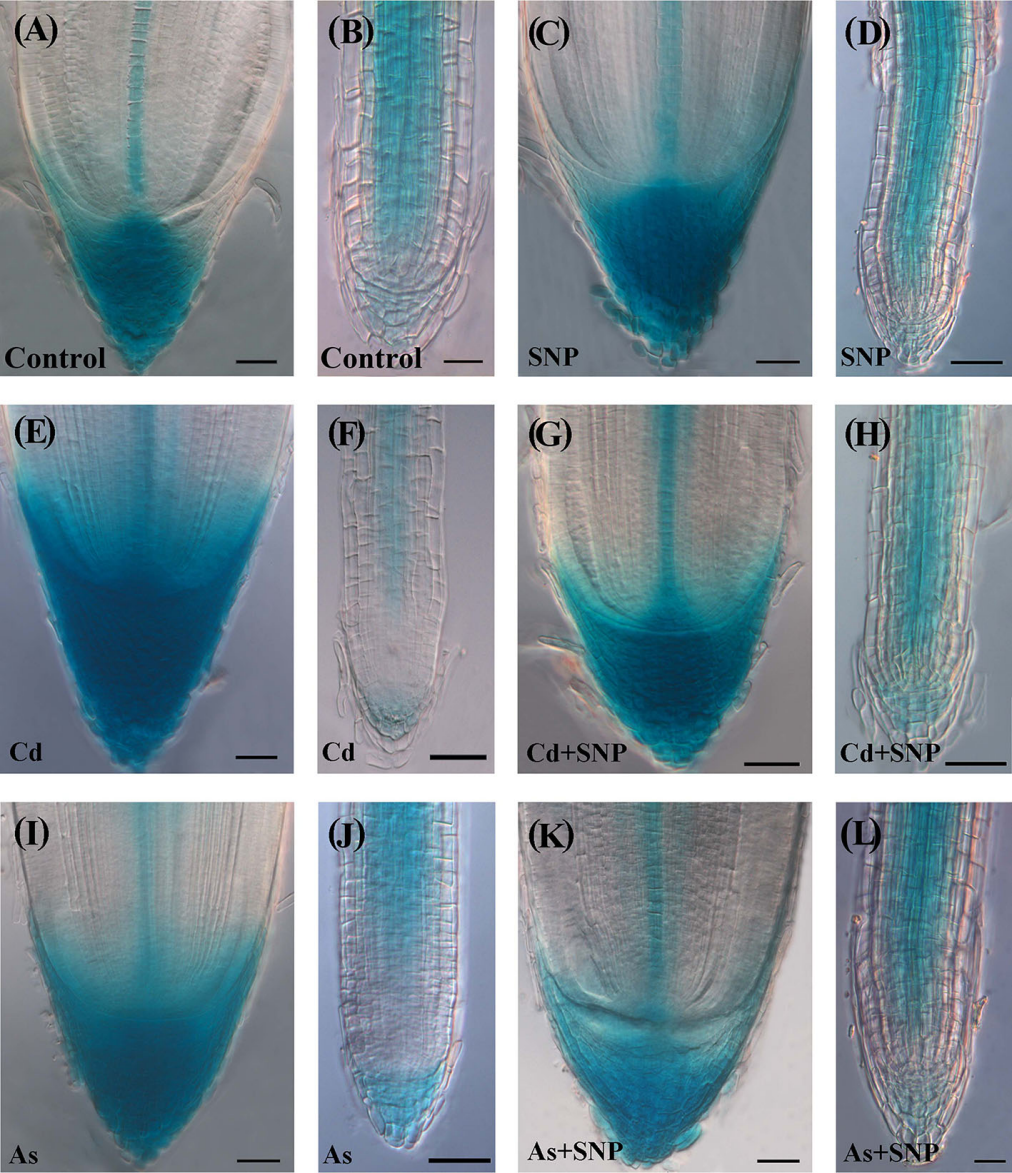
Expression pattern of *DR5::GUS* in adventitious roots **(A, C, E, G, I, K)**, lateral roots **(B, D, F, H, J, L)** of *Oryza sativa* *DR5::GUS* seedlings non-exposed (Control, **A, B**) or exposed to 50 µM sodium nitroprusside (SNP, **C, D**), 100 μM CdSO_4_ (Cd, **E, F**), 100 µM Na_2_HAsO_4_·7H_2_O (As, **I, J**), alone or combined (Cd + SNP, **G, H**, As + SNP, **K, L**). Bars: 20 µm **(B, L)**; 40 µm **(A, C–K)**.

The IAA distribution and accumulation were evaluated in the root system of *OsDR5::GUS* seedlings. No artefact in the intensity of the GUS signal due to the membrane injury caused by the pollutants (**Figure 1**) occurred because the auxin levels (**Figure 6**) matched with the GUS staining (**Figure 8** and **Supplementary Figure S3**).

In the Control-treatment, the GUS signal in the AR-apex was high in all cap cells, quiescent center (QC), and central procambium (**Figure 8A**). In the LRs, the signal was weak in the cap and apical meristem cells; by contrast, all cell files coming from the QC showed a consistent expression, except for the forming rhizodermis and exodermis (**Figure 8B**). The treatment with SNP alone did not result into significant changes in the localization/intensity of the auxin signal in the AR in comparison with the Control (**Figures 8A, C**). The LRs also showed the same expression pattern of the corresponding root type in the Control (**Figures 8B, D**). In the presence of Cd alone, a strong auxin signal continued to be shown by the cap cells of the ARs but differently from the two previous treatments, also by a wide part of the apical dome (**Figures 8A, C, E**). By contrast, at both apex and base, the LRs showed a signal lower than the corresponding roots of the previous treatments and a more extended apical zone without signal (**Figures 8B, D, F**). In the presence of Cd + SNP, the auxin signal in the AR apex was reduced with respect to Cd-alone treatment (**Figures 8E, G**) and was similar to the Control/SNP treatments (**Figures 8A, C, G**). In the LRs, the signal was instead reinforced in comparison with the Cd-alone-treatment,and with cellular localization as in the Control and SNP (**Figures 8B, D, H**). Under As alone, the apex of the ARs showed an intense auxin signal and with a wider extension in comparison with Control/SNP alone, but with reduced extension with respect to Cd alone (**Figures 8E, I**). However, in contrast with Cd, As reinforced the auxin signal in the LRs (**Figures 8F, J**), comparably with the Control/SNP treatments (**Figures 8B, D, J**). When As was combined with SNP, the pattern of auxin distribution in the ARs and LRs was as in the Control and Cd + SNP (**Figures 8A, B, G, H, K, L**).

In the AR-region of primary structure, the auxin signal was shown by all tissues except the rhizodermis, and in all treatments, with higher intensity in Cd or As alone treatments (**Supplementary Figures S3A–F**). During early development, the LRP was always totally expressed but, at further stages, the expression became progressively restricted to the primordium apical and basal parts, without evident differences among the treatments (**Supplementary Figures S3G–L**).

### Exogenous NO Enhances the Root Expression of *AUX1* Auxin-Influx Gene and Mainly in As Presence

**Figure 9:**
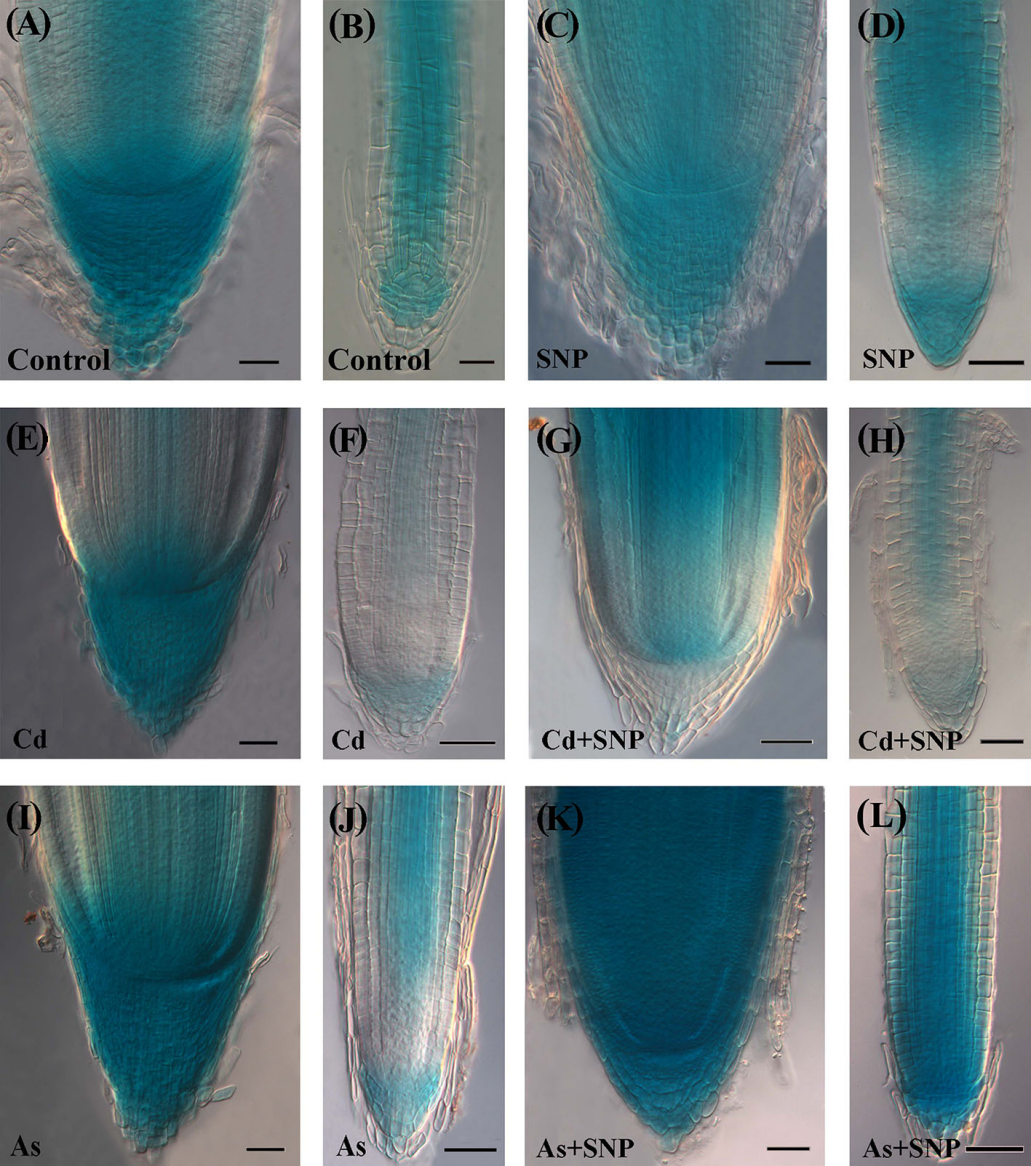
Expression pattern of *AUX1::GUS* in adventitious roots **(A, C, E, G, I, K)**, lateral roots **(B, D, F, H, J, L)** of *Oryza sativa* *AUX1::GUS* seedlings non-exposed (Control, **A, B**) or exposed to 50 µM sodium nitroprusside (SNP, **C, D**), 100 μM CdSO_4_ (Cd, **E, F**), 100 µM Na_2_HAsO_4_·7H_2_O (As, **I, J**), alone or combined (Cd + SNP, **G–H**, As + SNP, **K, L**). Bars: 20 µm **(B)**; 40 µm **(A, C–L)**.

In the Control the *AUX1::GUS* expression signal in the AR-apex was high in all cap, quiescent center (QC) with surrounding initials and procambium (**Figure 9A**). In the LRs, the signal was shown by the internal cap and all over in the apical meristem, continuing in the differentiating and mature tissues with the exception of rhizodermis and exodermis (**Figure 9B**). In the ARs and LRs, the treatment with SNP alone enhanced the diffusion of the *AUX1* signal in comparison with the Control treatment (**Figures 9A–D**).

In the treatment with Cd alone, a strong *AUX1* signal was shown by the cap cells of the ARs, as in the Control and SNP treatments, even if with a smaller extension (**Figures 9A, C, E**). In contrast with the AR, both the apex and the differentiated tissues of the LRs showed the lowest *AUX1* expression of all other treatments (**Figure 9F**). In the treatment with Cd + SNP, the intensity of the *AUX1* signal in the apex of the ARs was higher than with Cd alone, but the staining hardly occurred in the cap (**Figures 9E, G**). In the LRs, the *AUX1* signal remained weak in the apex, as with Cd alone but became higher at least in the central differentiating/differentiated tissues (**Figures 9F, H**). Under As alone, the apex of the ARs showed a very strong and extended *AUX1* signal (**Figure 9I**). In contrast with Cd alone, As reinforced the *AUX1* signal in the LRs (**Figures 9F, J**). When As was combined with SNP, the *AUX1* expression pattern/signal intensity in the apex of the ARs and LRs became incredibly high in all the cells (**Figures 9K, L**).

In the AR-region of primary structure, the *AUX1* signal was shown in all treatments at higher levels than in the Control but it extended to the rhizodermis only under As- and As + SNP-treatments (**Supplementary Figures S4A–F**). Starting from the inception, the LRPs showed the *AUX1* signal under all treatments; however, during and after protrusion, the expression signal decreased in the presence of Cd alone and highly increased with SNP treatments (**Supplementary Figures S4G–L**).

## Discussion

Results reveal that the interaction between auxins (IAA/IBA) and the pollutants (Cd or As) is mediated by NO in the rice root system with the LRs as the main target of pollutant-action and of the NO-mitigating reaction, but with differences between pollutants and auxins. In fact, cell membrane injury and the hydrogen peroxide formation, induced by both pollutants, are counteracted by exogenous NO only in the case of Cd, and the endogenous IAA levels are enhanced by both, but those of IBA only by As. Nonetheless, for both pollutants, the addition of the exogenous NO results into a reduction of the levels of both endogenous auxins and into a similar auxin localization in the root apices. In addition, both exogenous auxins, when added with each pollutant at 10 µM, exhibit the same behavior by counteracting the NO decrease caused by Cd and by unaffecting that caused by As.

It has been recently demonstrated that a different behavior of NO modulates the rice root-system response to the toxicity of Cd and As and results into a NO-mitigation of Cd damages only (Piacentini et al., 2020). By contrast, and by the use of the same rice cultivar and concentrations of the pollutants and the NO-donor, present data show a more complex scenario, in which the root system reaction to Cd, but also to As toxicity, involves IAA, and its precursor IBA, and their cooperation with the NO signaling molecule.

### Auxin as a Common Focus of Both NO Mitigating-Action and Pollutant Stress-Action

In rice, *OsASA2*, an early gene of the tryptophan-dependent IAA-biosynthesis and *OsYUCCA1*, one of the *YUCCA*-family genes involved in a late step of IAA-biosynthesis, have been reported as stress-related auxin genes (Tozawa et al., 2001; Sun et al., 2017). Present data show that the two genes were differently affected by the pollutants, with *ASA2* expression enhanced by As and not by Cd, and *OsYUCCA1* expression reduced by both pollutants (**Figure 7**). This suggests that the rice root system response to As involves an increase in IAA through ASA2, as a specific auxin-biosynthesis protein of the reaction to As and in accordance with its known upregulation by abiotic stresses in the same plant (Du et al., 2013). Also, when the rice seedlings are grown under different conditions for germination (continuous darkness), As enhances *ASA2* expression, differently from what happens with Cd (Ronzan et al., 2018).

Interestingly, the application of the NO-donor SNP together with As resulted in a reduction of *ASA2* expression and IAA levels (**Figures 6A** and **7**), suggesting a mitigating effect of the over-produced NO on the root stress-reaction to As involving a decrease in auxin biosynthesis. Present data also show that when NO was over-produced by SNP, alone or combined with the pollutants, a strong reduction of *YUCCA1* expression occurred, suggesting that *OsYUCCA1* expression was sensitive to the stress, and that also NO over-production might act as a stressor depending on the target gene (**Figure 7**). In accordance, an increase in oxidative damage, monitored as hydrogen peroxide production, occurred with SNP alone in comparison with the untreated control (**Figure 2**). It is possible that other member(s) of the *OsYUCCA* family (Zhang et al., 2018) is/are more specifically sensitive to either pollutant or to the mitigating NO-effect. For example, *OsYUCCA2* expression is enhanced by Cd and not by As at the same concentrations here used (Ronzan et al., 2018). In accordance with the hypothesis that other YUCCAs are involved, the IAA levels increased in the roots also after Cd treatment (**Figure 6A**).

It is known that IAA levels increase not only after enhanced biosynthesis, but also after the conversion of the auxin-precursor IBA into IAA (Schlicht et al., 2013). Our data show that IBA was present in the 10-days-old roots but at very low levels in comparison with IAA (**Figures 6A, B**), with this suggesting a conversion during the seedling growth period to enhance IAA levels, in accordance with what occurs in Arabidopsis dark-grown seedlings during root system formation (Veloccia et al., 2016).

Two main points, in apparent contrast each other, emerge from the *Results*. The first one is that Cd/As induces increases in IAA levels (**Figure 6**). The second one is that these IAA increases do not result into an increased LR formation but instead, into a decrease (**Figures 4B, D**), even if it is widely known that auxins are LR inducers (Du and Scheres, 2018). The first point is in contrast with a wide part of literature showing that these pollutants decrease auxin levels (Hu et al., 2013; Srivastava et al., 2013); however, the reverse is also possible. For example, Cd and As increase auxin biosynthesis and accumulation in rice roots in the time interval from two to ten days of seedlings grown (Ronzan et al., 2019) and Cd in the root-forming hypocotyl of Arabidopsis seedlings (Fattorini et al., 2017a). About the second point, the failure of LR formation by high endogenous auxin levels caused by the pollutants and/or a too high exogenous auxin input (**Supplementary Figure S1**) may be related to the observed altered cellular distribution and influx of auxin by AUX1 in the ARs (**Figures 8** and **9**). In the AR pericycle and endodermal cells competent for LR initiation (Ni et al., 2014) under normal endogenous auxin, these alterations may cause an alternative, but equally auxin-induced, morphogenic program, *i.e.* the ectopic formation of xylary cells, which may compete with rooting. In fact, auxin is a pleiotropic phytohormone activating a lot of morphoanatomical events in alternative to LRs (Fattorini et al., 2017b), acting alone or with the cooperation of other phytohormones, which can affect its levels and target molecules. For example, in Arabidopsis, ethylene (ET) and jasmonates (JAs) interact with auxin to form ectopic xylary cells instead of LRs starting from the same hypocotyl pericycle initial cells (Fattorini et al., 2017b; Fattorini et al., 2018), with the auxin influx carrier AUX1 controlling the switching between the two programs (Della Rovere et al., 2015). On the other hand, Cd is known to increase the formation of lignified cells either as a consequence of its toxicity or as an elicited defense response (Loix et al., 2017). In accordance, our preliminary histological observations on rice ARs show that ectopic xylary cell formation is caused by Cd or As as an alternative program to lateral rooting.

Another important point to understand is how the high endogenous auxin formed in the presence of Cd or As is associated with low NO levels, whereas the exogenous IAA application induces high NO levels (**Figure 5**). We show that the pollutants cause stress resulting into membrane injury (**Figure 1**) in accordance with data of the rice shoot treated with Cd (Singh and Shah, 2014) or As (Begum et al., 2016), and into hydrogen peroxide formation (**Figure 2**), and synthesis of ET according to our preliminary data. A fine-tuning of ROS and ET in stress responses is widely known (Zhang et al., 2016). Moreover, in sunflower seedlings a stable complex is formed between NO and the ET-biosynthesizing enzyme-ACC oxidase in response to salt stress (Singh and Bhatla, 2018). The same might occur in rice seedlings justifying the observed reduction in NO-epifluorescence signal (**Figure 5**) because the fluorochrome only binds to the free forms NO. It is clear that Cd/As may trigger this stress response, but not auxin *per se*, at least at the used concentration. In fact, exogenous IAA caused only minor changes in hydrogen peroxide formation in comparison with the Control (**Figure 2**), possibly unable to activate a NO/ET cross-talk, with the consequence that the NO fluorescence signal remained high (**Figure 5**). It is also possible that the increase in NO-epifluorescence levels after the exogenous application of IAA may be due to an increase in the endogenous IAA levels during the root growth period also resulting from the conversion of the endogenous IBA into IAA and the NO-formation coming from this conversion (Schlicht et al., 2013). In any case, the reduction of the NO-fluorescence signal caused by each pollutant reinforces the hypothesis that NO, at the tested condition, is a counteracting-factor in plant reactions to pollutant toxicity, with auxin as a common focus of both NO mitigating-action and pollutants stress-action (**Figure 5H**).

Interestingly, the IAA tissue-distribution and cellular influx in the roots (**Figures 8** and **9**) highlight a new result: a buffering role of NO on the auxin distribution/influx alterations induced by the pollutants, and in both the types of developed roots (ARs and LRs).

The fibrous root system of rice is formed by different root types, *i.e.*, the embryonic-in-origin ARs with an indeterminate growth pattern (Rebouillat et al., 2009) and the post-embryonic LRs formed by the ARs, and with a prevalent determinate growth pattern. In the roots with indeterminate growth, an auxin maximum is established at the apex and is essential for the defining and functioning of the stem cell niche from which the root growth depends (Della Rovere et al., 2016 and references therein). Present data show that both pollutants reduced AR-length (**Figure 4**), confirming previous results (Ronzan et al., 2018). They also show that this occurred by enhancing the cell population of the AR-apex with NO signal (**Supplementary Figure S2**) and with signals for auxin presence, monitored by the *DR5::GUS* construct, and auxin influx, monitored by *AUX1* expression (**Figures 8** and **9**). The consequence was an extending of the auxin maximum in the apex altering the stem cell niche (Ni et al., 2014), with negative consequences in its activity for the AR-growth. In accordance, in Arabidopsis a low NO apical level results in root elongation, whereas a higher NO level causes the opposite effect, also affecting auxin accumulation (Liu et al., 2017), and Cd and As negatively affect the identity of the QC in the stem cell niche (Fattorini et al., 2017a).

The LRPs were not particularly affected by the pollutants, at least in terms of hydrogen peroxide levels, always high (**Figure 2**), and of endogenous auxin influx, maximum definition, and basal and apical localization (**Supplementary Figures S3** and **S4**), with these events possibly explaining the observed increase in primordia formation (**Figure 4**) as an adaptative compensation to the AR-reduction in length, as also suggested for other abiotic stresses, *e.g.*, iron-deficiency (Sun et al., 2017). However, the total number of LRPs and LRs was highly reduced by both Cd and As (**Figure 4**) because the LRs were their common target, but with contrasting auxin-unbalancing effects. In fact, Cd highly reduced the endogenous auxin presence and influx in the LR apical region, whereas As increased it (**Figures 8** and **9**). Interestingly the treatment with the exogenous donor of NO caused *per se* an increase in *AUX1* expression, suggesting that the role(s) of NO on auxin influx needs further investigation.

In conclusion, NO is a signaling molecule able to cooperate with the two main auxins of the plant, IAA, and its precursor IBA, for mitigating the alterations caused to the root system by the toxic soil pollutants Cd and As. It seems to act indirectly and downstream of auxin and perhaps with the cooperation of other phytohormones. Interestingly NO mitigates Cd/As effects by reducing IAA levels. Its overproduction by the application of its donor-compound SNP enhances ROS formation (*e.g.* hydrogen peroxide, **Figure 2**) and superoxide anion (Piacentini et al., 2020). This suggests that NO effect is linked to the ROS pathway, with ROS causing IAA oxidation and consequent reduction of the IAA levels, thus determining a re-equilibrium of auxin homeostasis favouring LR induction.

## Data Availability Statement

All datasets presented in this study are included in the article/**Supplementary Material**.

## Author Contributions

DP designed, collected data, and performed the statistical analysis. FDR was involved in the acquisition and statistical analysis of data and in drafting the manuscript. LF performed the electrolyte leakage experiments and contributed to the discussion of data. AS carried out the quantitative determinations of auxins. GF and MA analyzed and interpreted the data, and wrote the manuscript, and MA revised it critically. All authors contributed to the article and approved the submitted version.

## Funding

This work was supported by Progetti per Avvio alla Ricerca, Sapienza University of Rome (AR118163F8C319B7) to DP and by Progetti di Ricerca Medi, Sapienza University of Rome (RM11916B558D1F93) to FDR.

## Conflict of Interest

The authors declare that the research was conducted in the absence of any commercial or financial relationships that could be construed as a potential conflict of interest.
