# Supplementary figures and images for: Nitric Oxide Cooperates With Auxin to Mitigate the Alterations in the Root System Caused by Cadmium and Arsenic
Source: Front Plant Sci. 2020 Aug 5;11:1182. doi: 10.3389/fpls.2020.01182 (PMC7419627; doi:10.3389/fpls.2020.01182)

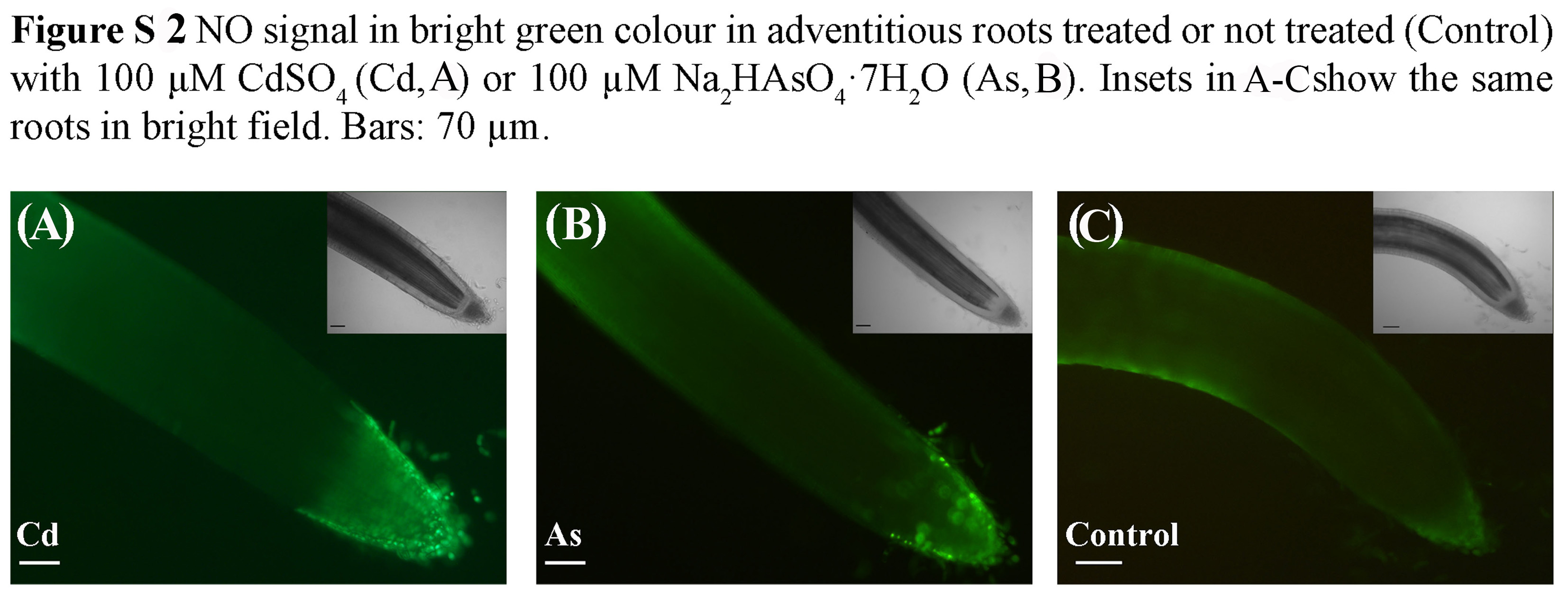

Supplement: Supplementary file 1 [file Image_1.jpeg]

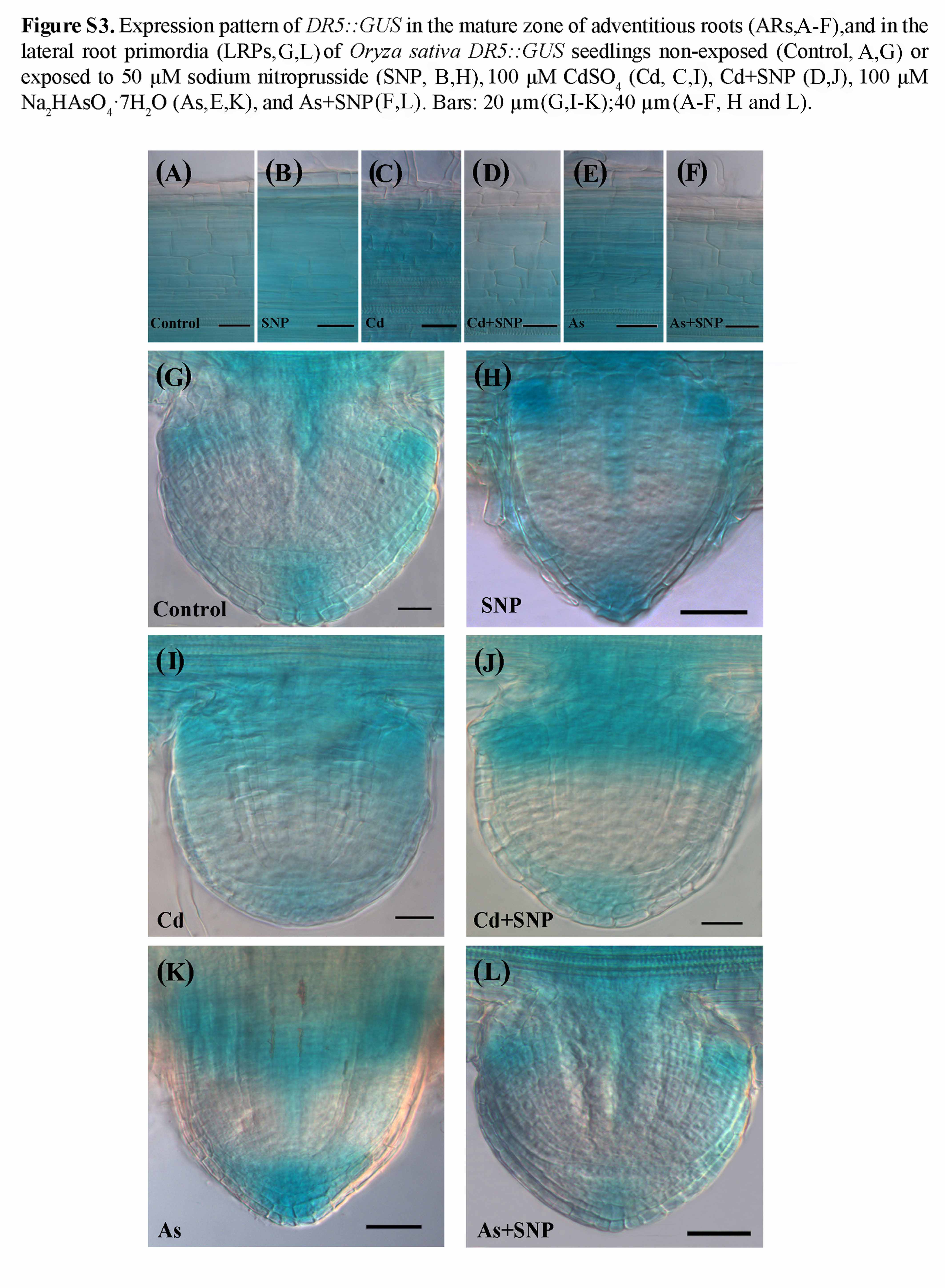

Supplement: Supplementary file 2 [file Image_2.jpeg]

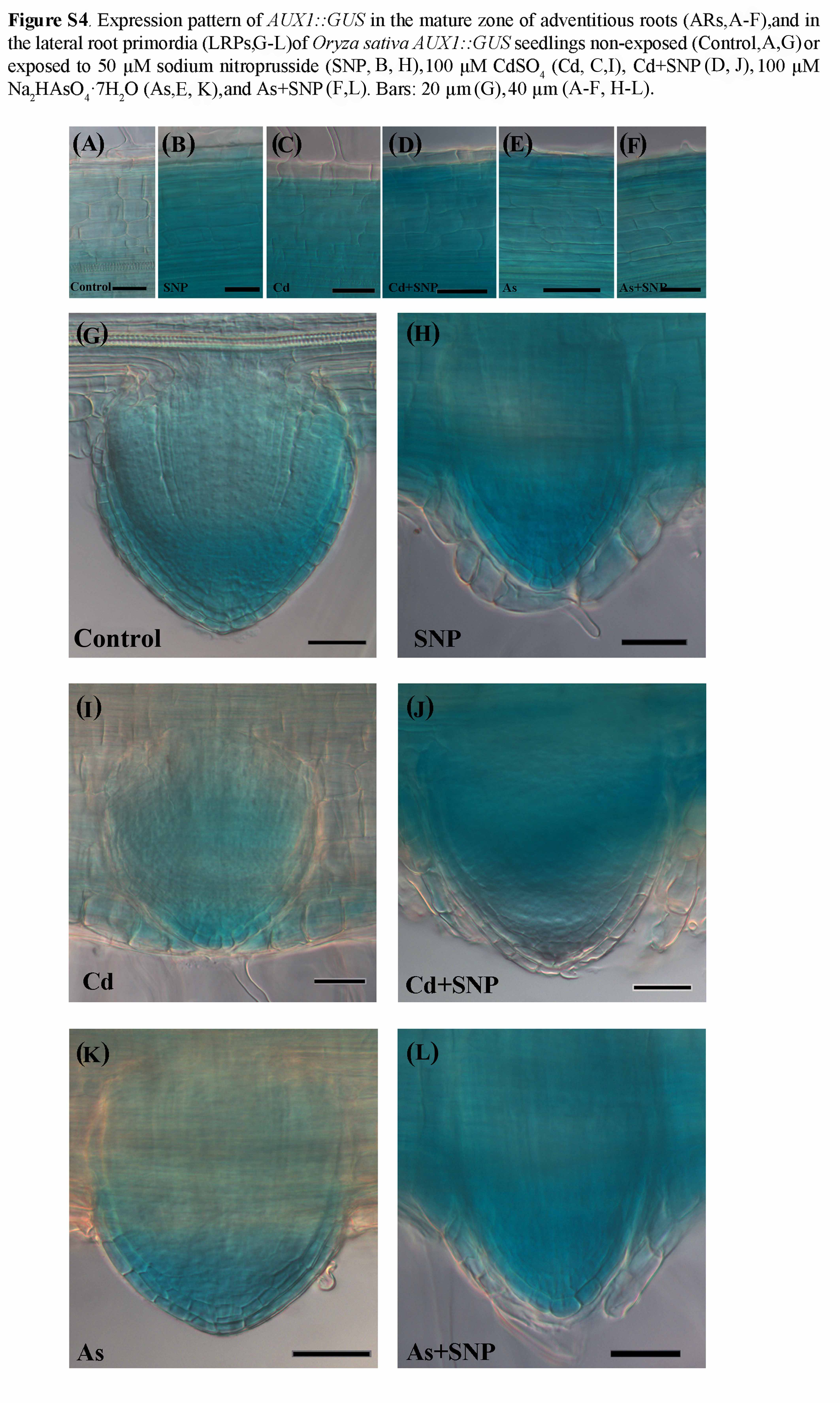

Supplement: Supplementary file 3 [file Image_3.jpeg]

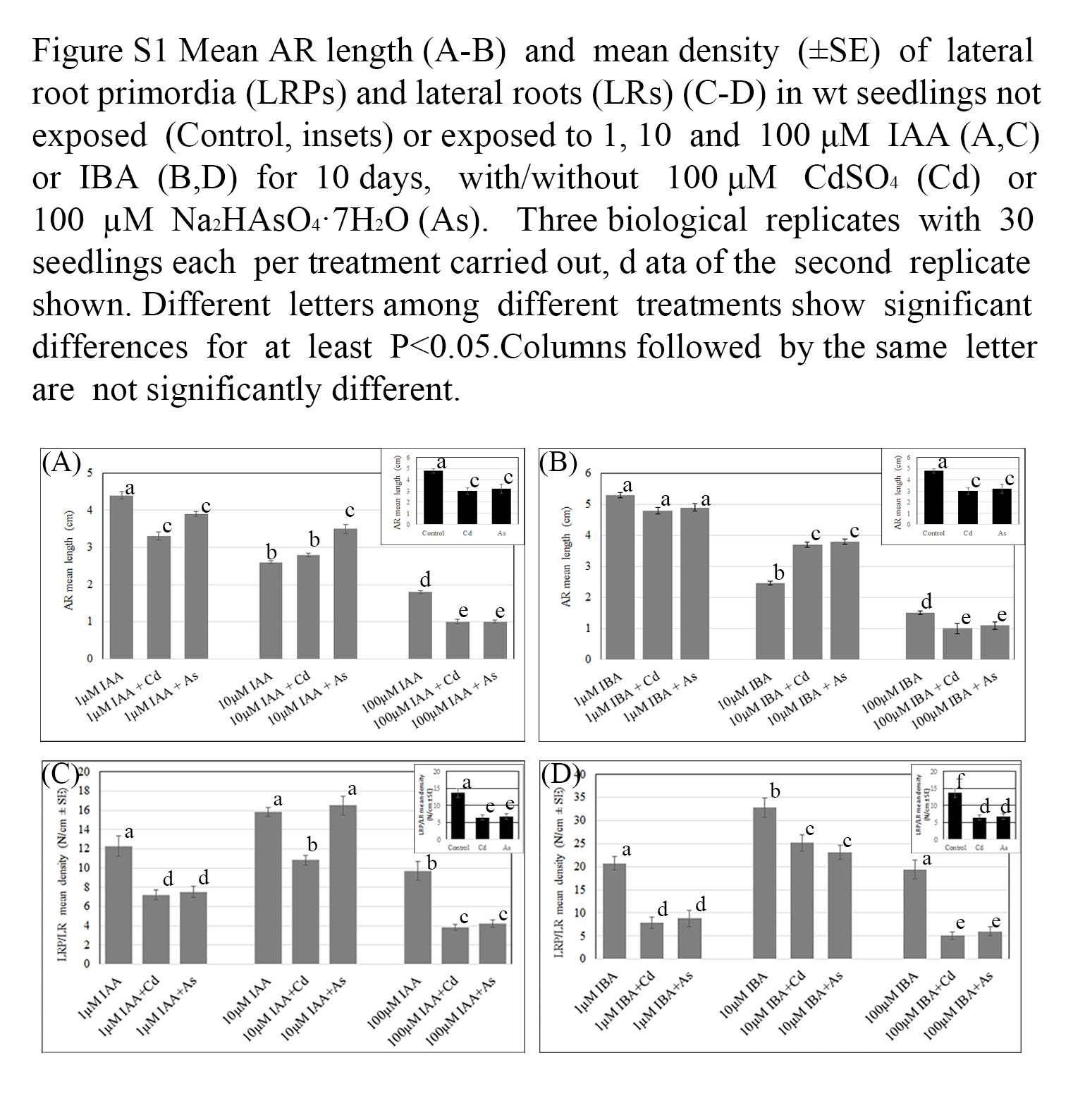

Supplement: Supplementary file 4 [file Image_4.jpeg]
