# Supplementary material for: Nitric Oxide Cooperates With Auxin to Mitigate the Alterations in the Root System Caused by Cadmium and Arsenic
Source: Front Plant Sci. 2020 Aug 5;11:1182. doi: 10.3389/fpls.2020.01182 (PMC7419627; doi:10.3389/fpls.2020.01182)
Supplement: Supplementary file 5 [file Table_1.pdf]

Table S1. List of PCR primers and accession numbers of the genes used in the study.

Gene Name	Accession number	Sequence (5' to 3')
YUCCA1	OSNPB_010645400	CCCTCAACGTCGCCAAAGA
		GCAGATCGGTCCCTTTCTCT
ASA2	OSNPB_030264400	AGCCACAACACGATGTACTCA
		ACATGCAGCGAGAAGAACCA
OsUBQ5 (housekeeping gene)	OSNPB_010328400	TTCTACAAGGTGGACGACGC
		AAAGAACAGGAGCCTACGCC
OsGAPDH (housekeeping gene)	OSNPB_040459500	CTGATGATATGGACCTGAGTCTACTTTT
		CAACTGCACTGGACGGCTTA
OsActin-1 (housekeeping gene)	OSNPB_030718100	TCTTGGCATCTCTCAGCACAT
		TGGCTTAGCATTCTTGGGTCC
